# Reversible protein assemblies in the proteostasis network in health and disease

**DOI:** 10.3389/fmolb.2023.1155521

**Published:** 2023-03-20

**Authors:** Verena Kohler, Claes Andréasson

**Affiliations:** ^1^ Institute of Molecular Biosciences, University of Graz, Graz, Austria; ^2^ Department of Molecular Biosciences, Stockholm University, Stockholm, Sweden

**Keywords:** phase separation, biomolecular condensate, aggregate, Hsp70, Hsp100, disaggregation, refolding, degradation

## Abstract

While proteins populating their native conformations constitute the functional entities of cells, protein aggregates are traditionally associated with cellular dysfunction, stress and disease. During recent years, it has become clear that large aggregate-like protein condensates formed *via* liquid-liquid phase separation age into more solid aggregate-like particles that harbor misfolded proteins and are decorated by protein quality control factors. The constituent proteins of the condensates/aggregates are disentangled by protein disaggregation systems mainly based on Hsp70 and AAA ATPase Hsp100 chaperones prior to their handover to refolding and degradation systems. Here, we discuss the functional roles that condensate formation/aggregation and disaggregation play in protein quality control to maintain proteostasis and why it matters for understanding health and disease.

## 1 Introduction

**FIGURE 1:**
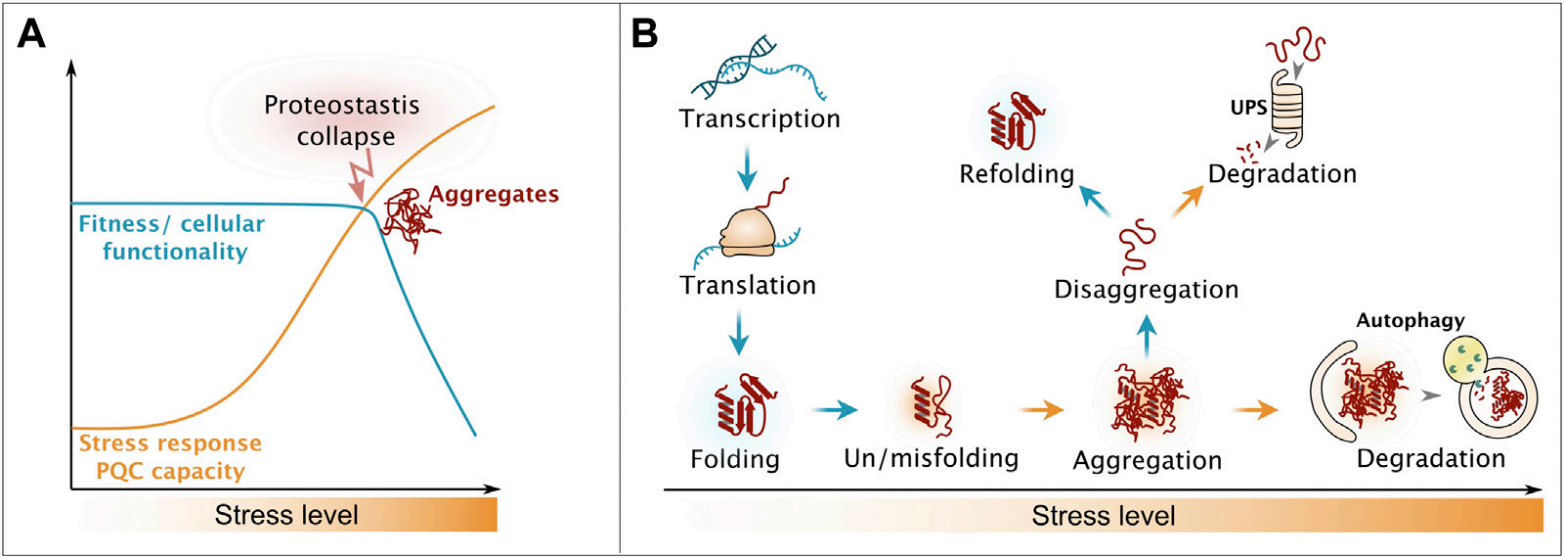
Influence of proteostatic stress on cellular fitness. **(A)** Upon increase of proteotoxic stress, cells activate stress response regimes, ensuring on keeping cellular processes functional. Overburden results in the collapse of the proteostasis system and occurrence of aggregates, leading to drastically decreased cellular healthspan. **(B)** Strategies of the cellular protein quality control system to counteract proteotoxic stress, end in the triage decision between refolding and degradation. Please see main text for details.

The cellular proteome encompasses proteins that populate a plethora of conformations, from stably folded proteins to those without fixed tertiary structures, consisting completely of intrinsically-disordered regions (IDRs). For example, in human cells the majority of proteins carry both folded domains and IDRs, while 37% of the proteome is fully folded and only 5% of the proteins occupy entirely disordered conformations (Tsang et al., 2020). The conformational complexity is increased by the occurrence of higher-oligomeric protein assemblies that are formed by both stochastic interactions as well as by direct interactions regulated by internal and external cues. These assemblies may form by liquid-liquid phase separation (LLPS) and it is now established that such biomolecular condensates have functional roles (Banani et al., 2017). In contrast, protein aggregates traditionally have been viewed as disordered structures, spontaneously formed by misfolded proteins (Wang and Roberts, 2018). While biomolecular condensates are essential for cellular regulation, the occurrence of protein aggregates is generally a sign of a structurally compromised proteome, and typically associated with cellular stress (Figure 1A). However, it is nearly impossible to strictly categorize LLPS and protein aggregation as fully separate processes (Lee et al., 2016; Boeynaems et al., 2017). Furthermore, protein aggregation as well as LLPS are accelerated by stress indicating that the processes are closely linked and involved in management of an overloaded protein quality control (PQC) system.

Cellular protein homeostasis (proteostasis) is constantly challenged by the flow of unfolded proteins produced by protein biosynthesis as well as from protein misfolding caused by stochastic errors in gene expression and damage from proteotoxic stressors including but not limited to heat, oxidants and metabolic imbalances (Figure 1B) (Zakariya et al., 2022). Chaperones are in place to assist on-pathway protein folding and thereby to suppress the buildup of aberrant misfolded proteins (Balchin et al., 2020). Here, the class of chaperones belonging to heat shock proteins (Hsps) of the Hsp70 family has a key function. Hsp70s are found across all kingdoms of life and operate in nearly all organelles in eukaryotic cells. Details on their ATPase-driven mode of action is extensively described in recent reviews (Rosenzweig et al., 2019; Kohler and Andréasson, 2020). Stress damage may overwhelm the chaperone folding machinery causing proteotoxic misfolded proteins to assemble into higher-oligomeric states, including aggregates (Mogk et al., 2018). Thus, cells sequester these potentially toxic protein species into quality control compartments by utilizing small heat shock proteins that act as aggregation-promoting factors (aggregases) and thus protect the proteome from aberrant interactions (Haslbeck et al., 2019). During recovery from stress, disaggregation machineries promote the disentanglement of the aggregates and lead to recovery of the constituent proteins (Mogk et al., 2018; Nillegoda et al., 2018). Metazoan cells differ drastically from plant, fungi and bacteria in respect to their disaggregation machinery, which will be explained in greater details in later sections. Once aggregated proteins are disentangled, their refolding is considered to be the preferred pathway, yet alternative pathways involve complete removal from the cell by degradation (Wallace et al., 2015; Määttä et al., 2020). The Hsp70 chaperone network aids both the refolding and targeting to the two major proteolytic systems, the ubiquitin-proteasome system (UPS) and the lysosomal/vacuolar system *via* autophagy (Figure 1B) (Wallace et al., 2015; Määttä et al., 2020).

In this review, we aim to give an overview on higher-order assembly processes and their interplay. We will discuss the cellular and organismal consequences of disturbances of these intricately balanced systems in response to internal and external cues. A main focus is on the cellular strategies that regulate these processes in metazoan and model organisms, with a special emphasis on disaggregation, a process that determines partitioning between refolding and degradation faiths.

## 2 Similarities and differences of biomolecular condensates and aggregates

**FIGURE 2:**
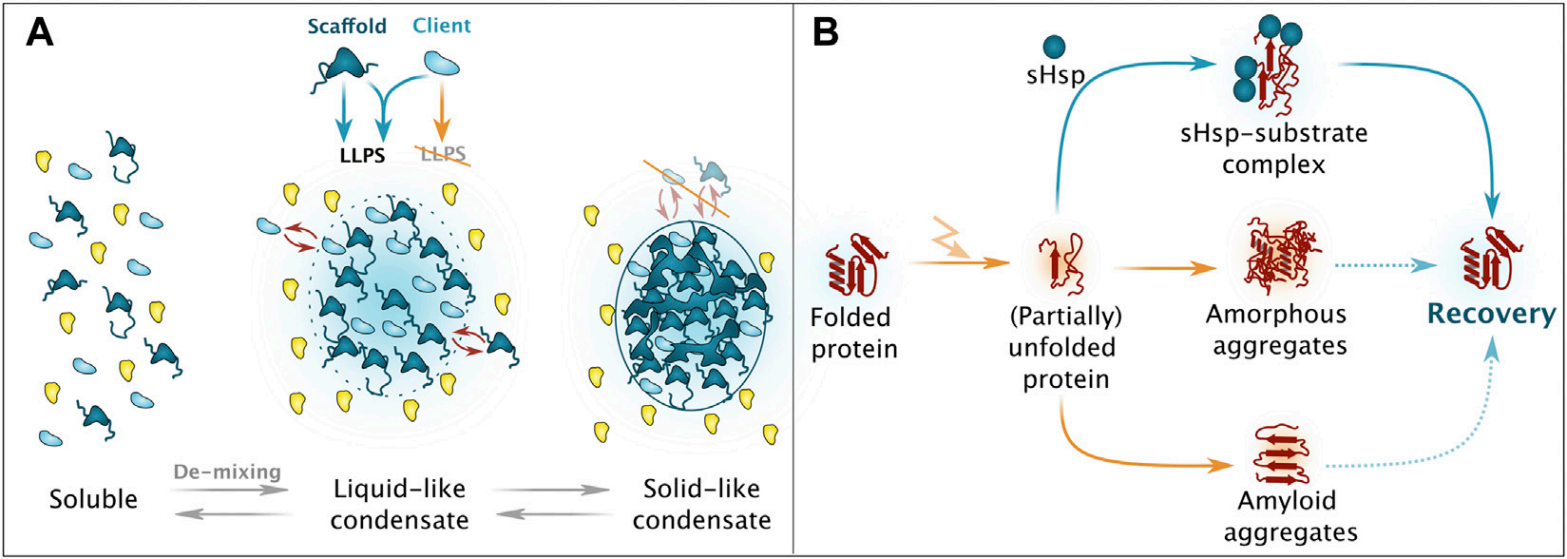
Simplified overview on phase separation and aggregation. **(A)** Upon presence of scaffold proteins with potent liquid-liquid phase separation (LLPS)-enabling features, de-mixing of a protein solution can take place, eventuation in liquid-like condensates, consisting of both scaffold (dark blue) and client (light blue) proteins. Ageing processes result in solid-like condensates that no longer support dynamic exchange of constituents with the surrounding milieu. Please note that also chaperones and protein quality control factors are involved in these processes. **(B)** Unfolded resp. partially unfolded protein species can adopt different aggregate forms. Depending on the intrinsic nature, amorphous as well as amyloid aggregates can occur, with a limited potential for recovery. Small heats hock proteins (sHsps) are responsible for targeted and regulated sequestration and alleviate subsequent recovery/refolding.

Aiming for a simplified definition, generally accepted features applicable for most biomolecular condensates generated by LLPS include the reversibility of the dynamic phase-separated assemblies at distinct stages and the fact that their protein constituents generally maintain their tertiary structures (Figure 2A) (Alberti and Hyman, 2021; Mehta and Zhang, 2022). On the other hand, aggregated proteins occupy aberrant folds and strictly require the assistance from PQC factors for disentanglement and re-folding (Figure 2B) (Zhao et al., 2020). However, phase-separated assemblies precede aggregate formation and some condensates also require assistance for reversal under specific circumstances, this includes condensate hardening (Lee et al., 2016; Boeynaems et al., 2017), which will be discussed in later sections.

### 2.1 Phase separation enables dynamic and efficient regulation

Biomolecular condensates or liquid droplets are dynamic and reversible assemblies of molecules that can be dissolved and reused immediately. They assemble *via* LLPS and the molecules are concentrated in a liquid-like compartment that persist in the surrounding milieu (Brangwynne et al., 2011; Brangwynne et al., 2009; Li et al., 2012; Aggarwal et al., 2013; Feric and Brangwynne, 2013; Wippich et al., 2013). LLPS spatially restricts movement of the molecules without fully trapping them and thus enables controlled recruitment and release kinetics (Zhao et al., 2020).

Their biogenesis and structural integrity is exclusively based on protein-protein or protein-nucleic acid interactions and components of these membrane-less compartments connect and readily exchange with the external environment (Phair and Misteli, 2000). LLPS is driven by the minimization of global free energy, by maximizing weak inter- and intramolecular interactions between constituting macromolecules. This process only occurs at a certain solubility limit with a saturation concentration, which is characteristic for each phase-separating system (Alberti and Hyman, 2021). Whether the system undergoes LLPS not only depends on the molecular identity and solution concentration of the constituents and their post-translational modifications but also on the temperature as well as pH and concentrations of salt, ions, co-solutes, and small metabolites like ATP. RNAs are further key components driving the formation of biomolecular condensates and determine their composition and specificity (Brangwynne et al., 2009; Wolf et al., 2014; Munder et al., 2016; Patel et al., 2017; Hayes et al., 2018; Snead et al., 2019; Adame-Arana et al., 2020).

Condensates are generally composed of scaffolds and client proteins, the first harboring a high number of valences, acting as drivers of LLPS and also playing an essential role in the threshold concentration (Banani et al., 2016). Scaffolds are usually large, abundant proteins without enzymatic activity and condensate assembly depends on their ability to form a dense network of intermolecular interactions (Li et al., 2012). Key for the network are interactions provided by IDRs, which are low-complexity domains with biased amino acid compositions (Molliex et al., 2015; Nott et al., 2015; Patel et al., 2015; Wang et al., 2018). Client proteins have a lower interaction valence and are recruited to condensates formed by the scaffold factors. (Burke et al., 2015; Saha et al., 2016; Franzmann et al., 2018; Murthy et al., 2019; Guillén-Boixet et al., 2020). During LLPS, proteins typically do not undergo extensive structural changes and remain mostly disordered, as observed with NMR (Figure 2A) (Phair and Misteli, 2000).

Due to the lack of membranes, formation and disassembly of condensates *via* LLPS has the potential to respond rapidly to minor changes in the environment (Lin et al., 2015; Molliex et al., 2015; Nott et al., 2015). Their dynamic nature makes biomolecular condensates ideal compartments as biological reaction centers, signaling hubs and for organizing and regulating key biological processes such as splicing, translation, transcription and chromosome condensation (Su et al., 2016; Gueroussov et al., 2017; Tsang et al., 2019). The reported functions of biomolecular condensates include enhancement or suppression of biochemical reactions, buffering protein concentrations, detecting environmental changes and exertion of mechanical forces (Riback et al., 2017; Franzmann et al., 2018; Shin et al., 2018; Klosin et al., 2020). Biomolecular condensates are abundantly present in the cytoplasm, the nucleus, the mitochondrial matrix, the stroma of chloroplasts and the cytosol of bacteria (Zaslavsky and Uversky, 2018). Some structures are common to all cells, like nuclear speckles, paraspeckles and nucleoli, all found in the cellular nucleus, as well as cytoplasmic P-bodies and stress granules (Frottin et al., 2019; Ilık and Aktaş, 2022; Kedersha et al., 1999; Riggs et al., 2020; Sheth and Parker, 2003; Xing et al., n.d.).

Eukaryotic cells exposed to proteotoxic stressors require mechanisms that guarantee the integrity of the proteome until normal growth conditions are restored. Here, LLPS and molecular condensates play a central role. Key steps include global inhibition of protein synthesis and the selective upregulation of expression of stress response factors (Dever et al., 1992; Brostrom et al., 1996; Harding et al., 2003; Lu et al., 2004). The inhibition of translation and the subsequent rise of ribosome-free RNA in the cytosol is mainly regulated by stress-induced phosphorylation and inactivation of the translation initiation factor eIF2α. The released RNA and eIF2α are sequestered in stress granules *via* LLPS. When the stress is relieved, the stress granules shrink, eIF2α is dephosphorylated and mRNA translation is resumed. The coordinated reactivation of these abundant protein synthesis components is required for adaptive gene expression that trails minutes to a few hours behind the removal of the stress (Jousse et al., 2003; Wheeler et al., 2016).

### 2.2 Protein aggregation is a protective response to an overwhelmed proteostasis system

Protein aggregation is the association of misfolded proteins in higher-order assemblies, usually formed in cells with severely perturbed proteostasis, where the build-up of proteins with aberrant conformations exceeds PQC capacity. Aggregates consist of proteins at least in part in non-native states and involve stable intramolecular molecular interactions (Figure 2B). These non-native conformations are often toxic to a cell and are usually recognized by the cellular PQC system (Emma Mee Hayes et al., 2022). Practically all protein species are susceptible to aggregation, yet in the cell the process appears to affect proteins differentially. Proteins especially susceptible to aggregation were found to be large, have a high isoelectric point, disordered regions and hydrophilic amino acids (Kramer et al., 2012; Walther et al., 2015; Hosp et al., 2017; Uemura et al., 2018; Määttä et al., 2020).

Aggregates can be grouped into highly-ordered amyloid fibrils and disordered or amorphous protein aggregates, with alternative classifications based on size, reversibility of the aggregation, tertiary structure, modifications and morphology suggested in (Narhi et al., 2012). Some proteins misfold into a more ordered and less dynamic state such as the rigid amyloid fold (Serio et al., 2000). The amyloid conformation is typically not a functional state but a generic structural motif, consisting of elongated assemblies of nearly identical ß-sheets that are stacked onto each other. Amyloids are thermodynamically extremely stable, thus these conformations challenge the view that natively folded proteins populate the most stable conformations (Raabe et al., 2021). Moreover, amyloids may act as seeds converting other soluble proteins into the amyloid state, thus this aggregate subtype acts as a potentially infectious agent and may carry epigenetic traits (Serio et al., 2000; Raabe et al., 2021).

Small heat shock proteins (sHsps) and disaggregase machineries ensure that the aggregation is reversible despite the stable nature of protein aggregates [please see (Gallardo et al., 2021) for further details]. sHsps are regarded as first line of defense when cells experience proteotoxic stress by interacting with a wide plethora of substrates, forming stable sHsp-substrate complexes leading to their sequestration. These chaperones counteract uncontrolled aggregation of their substrates, but do not facilitate refolding upon stress relieve, a task left for cellular disaggregation machineries (extensively reviewed in (Mogk et al., 2019)). The reversibility is a protective mechanism (Saad et al., 2017) that may involve reactivation of the aggregated protein but extensively damaged proteins that cannot be repaired are ultimately subjected to degradation (Specht et al., 2011; Malinovska et al., 2012; Ungelenk et al., 2016).

The persistence of aberrantly folded and aggregated proteins is linked to cytotoxicity (Figure 1A). The proteotoxicity may involve loss of function of the trapped protein but has also been shown to be caused by co-sequestration and depletion of PQC factors essential for proteostasis, the disruption of cell membrane integrity due to aberrant interactions and perturbation of interactions/trafficking (Goggin et al., 2008; Olzscha et al., 2011; Arlet et al., 2014; Yu et al., 2014; Cenini et al., 2016; Grima et al., 2017). The aggregation may in turn be driven by pathological mutations, rendering proteins prone for misfolding, or accelerated by aging that leads to a global decline in PQC capacity due to decreased expression of chaperones and degradation factors and the overall accumulation of misfolded proteins caused by summation of external/internal cues (Hipp et al., 2019; Stein et al., 2022).

### 2.3 Biomolecular condensates fluently transition into aggregates

Biomolecular condensates initially show liquid-like properties and some of these phase-separated compartments age towards less dynamic gel-like or inert solid states (Molliex et al., 2015; Harmon et al., 2017; Riback et al., 2017; Iserman et al., 2020). Protein concentration, the absence of binding partners and a low water content enhance condensate aging and *vice versa* increased protein compositional heterogeneity in biomolecular condensates inhibits condensate hardening (Patel et al., 2015; Maharana et al., 2018; Majumdar et al., 2019). Condensate aging influences protein function, as a more liquid phase allows increased and more dynamic molecular interactions, while a more solid phase is difficult to reverse and sequesters proteins from the cytosol (Hernández-Vega et al., 2017). In yeast, the formation of solid-like condensates has been shown to be a physiological process serving an adaptive function. During stressful conditions, including heat shock, the downregulation of housekeeping protein synthesis allows yeast to reduce the burden of newly produced misfolding-prone proteins (Kroschwald et al., 2018; Kroschwald et al., 2015; Riback et al., 2017; Iserman et al., 2020). The translation initiation factor and stress granule component Ded1 is required for translation of mRNAs that encode housekeeping genes and is disposable from mRNAs encoding PQC factors. In response to heat stress, Ded1 phase-separates to form biomolecular condensates that harden quickly, leading to a situation where Ded1 is trapped inside stress granules. This in turn leads to changes in mRNA translation resulting in reduced expression of housekeeping genes and favored expression of stress factors. Dissipation of the hardened Ded1-condensates during stress recovery requires assistance from PQC factors (Kroschwald et al., 2018; Kroschwald et al., 2015; Riback et al., 2017; Iserman et al., 2020). Hence, the temperature sensitivity of Ded1 itself is suggested to determine global temperature sensitivity and the point, where yeast stops growing and instead invests into stress factor production.

LLPS is sensitive to stress-provoked changes, thus stress may lead to conformational alterations and/or reveal new interaction sites, potentially promoting condensate aging (Alberti and Hyman, 2021). Condensate-forming proteins carry long segments of IDRs that on the one hand function as scaffolds for condensate assembly and on the other hand render these proteins more misfolding- and aggregation-prone (Hegyi and Tompa, 2008; Määttä et al., 2020). Condensate hardening is associated with protection of biological function during stress, as the hardened state is proposed to act as kinetic trap for potentially aggregation-prone proteins, and thus hinders further damage caused by soluble misfolded proteins. Further, it relieves the burden from the PQC system (Riback et al., 2017; Franzmann et al., 2018; Kroschwald et al., 2018; Iserman et al., 2020).

Misfolding-prone proteins accumulate in biomolecular condensates and promote their hardening by interacting with other proteins in the condensates and by establishing long-lived physical crosslinks that also may include RNA (Choi et al., 2008; Lee et al., 2016; Boeynaems et al., 2017). Interestingly, defective ribosomal products, originating from ongoing translation and increasing upon stress and with age, are the main source of misfolding-prone proteins in cells and are also implicated in condensate aging (Schubert et al., 2000). They tend to accumulate in stress granules, nucleoli and PML bodies, another phase-separated compartment, and thus these assemblies are suggested to present overflow compartments that sequester misfolded proteins when their concentration reaches a critical level (Audas et al., 2016; Ganassi et al., 2016; Frottin et al., 2019; Mediani et al., 2019). In this line, occurence, formation and dissolution of biomolecular condensates like stress granules, have dual functions, they can serve as regulatory compartment to stop/re-activate translation according to cellular needs during exposure to stress (mentioned earlier in the text), but also represent storage compartments for unfolded proteins for easier chaperone-mediated downstream processes. Ubc9^ts^ is a misfolded model protein with a weakly destabilizing amino acid substitution and has been shown to become massively enriched in reconstituted condensates causing condensate aging and hardening (Mateju et al., 2017; Guillén-Boixet et al., 2020). Consistently, Ubc9^ts^, SOD1 and other misfolding-prone proteins were shown to accumulate in stress granules, changing their properties and dynamics (Ganassi et al., 2016; Lee et al., 2016; Boeynaems et al., 2017; Mateju et al., 2017; Apicco et al., 2018). Under native conditions, stress granules require RNA for assembly and enzymatic RNA removal leads to their disintegration (Bounedjah et al., 2014). Intriguingly, stress granules containing mutated SOD1 are resistant to RNA removal (Mateju et al., 2017). It was shown that misfolded proteins enter LLPS compartments to prevent their irreversible aggregation (Nollen et al., 2001) (Frottin et al., 2019). Biomolecular condensates might also change the kinetics of protein aggregation by promoting the formation of a rate-limiting nucleation point for protein misfolding and aggregation (Weber et al., 2019).

### 2.4 Disease-associated mutations are drivers of misfolding and aggregation

As biomolecular condensates play essential roles in cellular organization and physiology, failed LLPS can culminate in protein misfolding and aggregation (Babinchak and Surewicz, 2023). In line, anomalous LLPS and aberrant membrane-less compartments are associated with the pathogenesis of multiple human diseases. There is a strong link between condensate forming-proteins and age-related diseases such as neurodegeneration and cancer (Alberti and Hyman, 2021; Wang et al., 2021; Uversky, 2022). Proteins linked to neurodegeneration form condensates that promote protein aggregation and amyloid formation (Molliex et al., 2015; Patel et al., 2015; Ambadipudi et al., 2017; Wegmann et al., 2018; Babinchak et al., 2019; Ray et al., 2020). Many mutations are thought to change the conformational landscape of proteins, promoting amyloid-like interactions culminating in aggregation (Mackenzie et al., 2017; Murray et al., 2017; Qamar et al., 2018). For instance, mutations in proteins associated with Amyotrophic Lateral Sclerosis (ALS) lead to altered rates of condensate hardening (Midic et al., 2009; Murakami et al., 2015; Patel et al., 2015; Wang et al., 2018; Tsang et al., 2020). In pathological conditions, it is speculated that LLPS may even favor the formation of aggregates (Zbinden et al., 2020).

Insoluble protein deposits are hallmarks of neurodegenerative diseases, e.g., Alzheimer’s disease, Parkinson’s disease, Huntington’s disease and ALS. Aberrant aggregation involves a cascade of events and requires extended periods of time and eventually manifests in the clinical phase of neurodegeneration, thus the decline of neuronal health correlates with accumulation of aggregates (Hou et al., 2019). The disease-linked proteins Tau and α-Synuclein are inherently disordered and only obtain a defined structure when they associate with their binding partners (Avila et al., 2016; Stephens et al., 2019). Expanded repeat sequences such as polyQ, HTT and polyGA introduce unfolded protein stretches that promote protein aggregation (Nonaka et al., 2018; Bonfanti et al., 2019). During aggregation, the misfolded protein monomers come into contact, forming soluble conglomerates that further aggregate *via* structural rearrangements into stable and highly organized fibrils or amorphous aggregates without higher-ordered structure. Some studies ascribe oligomers to be the more toxic proteins species as they were shown to penetrate lipid bilayers and cause stress (Wegmann et al., 2016; De et al., 2019a; De et al., 2019b; Lobanova et al., 2022; Meng et al., 2022). Thus, the formation of aggregate fibrils, such as neurofibrillary tangles and Lewy-bodies, and the consequent sequestration is proposed to delay toxicity (Cowan and Mudher, 2013; Chartier and Duyckaerts, 2018). Interestingly, it was demonstrated that aggregate formation and progressive motor decline in a mouse model of Huntington’s disease depend on continuous expression of polyQ and thus can be reversible (Yamamoto et al., 2000).

## 3 Disaggregation is a decision point for refolding and degradation

To counteract aberrant condensate or aggregate formation, PQC factors are required to reset the stressed system. The transcriptional stress response system driven by heat shock transcription factors (HSFs) plays a key role. HSFs induce gene regulatory programs that support the removal of misfolded and aggregated proteins, thereby counteracting proteostasis collapse (Joutsen and Sistonen, 2019). In yeast it has been firmly established that the activity of Hsf1 is negatively regulated by levels of free Hsp70, with the result that misfolded proteins titrate Hsp70 to activate Hsf1 (Masser et al., 2020). Similarly, human HSF1 is negatively regulated by Hsp70 (Kmiecik et al., 2020; Masser et al., 2020). Thus, HSFs function as a proteostasis-sensitive mechanism that control PQC factor levels before and after stress.

Disaggregation is an essential first step to disentangle the aggregated protein and thus to either enable its subsequent rescue by refolding or removal by degradation. Most proteins in aggregates have been found to undergo disaggregation proportional to their aggregation propensity, i.e., a more severe loss in solubility is counteracted by faster disaggregation. It was recently demonstrated that proteins carrying IDRs are disaggregated faster *in vivo* (Määttä et al., 2020). On the molecular level, the explanation may simply be found in that weaker intramolecular interactions permit faster disentanglement. Alternatively, disordered regions that drive aggregation might also speed up subsequent extraction from aggregates by presenting flexible loop regions that the disaggregase machinery can bind. In this line, it was recently shown that *in vitro* disassembly of α-Synuclein fibrils requires N- and C-terminal extensions allowing chaperone binding cycles to facilitate the generation of power strokes (Gao et al., 2015).

**FIGURE 3:**
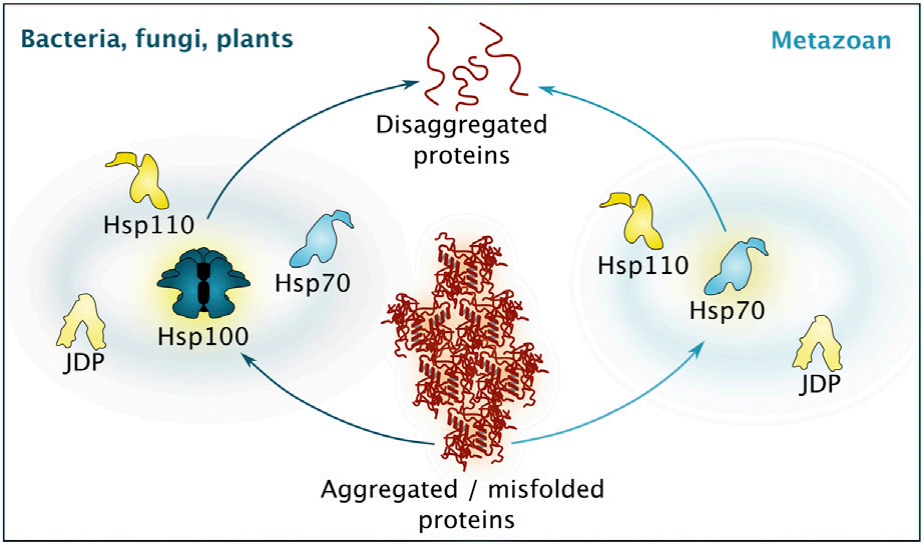
Simplified comparison between different disaggregation machineries. While bacteria, fungi and plants rely of disaggregation based on the activity of a Hsp100 isoform, supported by Hsp70, Hsp110 and a diverse set of J-domain proteins (JDP), metazoan disaggregation is based on Hsp70 activity, supported by co-chaperones belonging to the Hsp110 and JDP class. Please note that additional chaperones and protein quality control factors are involved to guarantee efficient disaggregation.

The disaggregation machinery itself is based on a conserved bi-chaperone disaggregase centered around Hsp100 family members and Hsp70. While plants, fungi and even bacteria carry the complete system, metazoans have lost the Hsp100 component and rely on a system powered by Hsp70 working in concert with J-domain protein (JDP) cochaperones and Hsp110 nucleotide exchange factors (NEFs) (simplified overview found in Figure 3). See brief description below, or for an in-depth description of different disaggregation mechanisms, see recent reviews (Nillegoda et al., 2018; Shorter and Southworth, 2019).

### 3.1 Cellular disaggregation and refolding strategies

Disaggregation and refolding of proteins trapped in aggregates is central to resetting the proteostasis system during proteotoxic stress recovery. This task is a complex and energy-consuming process, which involves unfolding of the aggregated species and then re-folding of the polypeptide into the native state. The process has parallels to *de novo* folding that occurs at the ribosomal tunnel exit but is not impacted by the strict directionality of mRNA translation since disaggregation can proceed from any end of a substrate. Briefly, the aggregates are handled by chaperone disaggregase machineries, initially characterized in yeast (Parsell et al., 1994; Glover and Lindquist, 1998; Mogk et al., 1999; Weibezahn et al., 2004). Notably, while, for example, bacteria, fungi, plants and protists possess a powerful bi-chaperone disaggregase centered around the ring-shaped AAA+ -chaperone Hsp100 (ClpB in bacteria, Hsp104 in yeast, Hsp101 in plants) and Hsp70, there is no cytosolic Hsp104 paralogue in metazoans (Sanchez and Lindquist, 1990; Parsell et al., 1994; Glover and Lindquist, 1998; Goloubinoff et al., 1999; Hong and Vierling, 2001; Mosser et al., 2004; Shorter, 2011).

Yeast Hsp104 cooperates with Hsp70 assisted by its JDP co-chaperones and Hsp110 class nucleotide exchange factors (NEFs) to thread trapped polypeptides in an ATP-dependent manner through its central pore, resolving a wide range of protein aggregates and hardened condensates (simplified overview found in Figure 3) (Deville et al., 2017; Gates et al., 2017; Kaimal et al., 2017). The role of Hsp70 is to recruit Hsp104 to the surface of the aggregate and to initiate disaggregation, likely by modifying the aggregate surface. Hsp70 in turn depends on its JDP to find its bindings sites and together they have the potential to disaggregation. Even though not required for disaggregation *in vitro* (Tessarz et al., 2008), yeast Hsp110 (Sse1 and Sse2) NEFs have been shown to be strictly required for this process *in vivo*, likely representing an indirect mechanism by resetting Hsp70 *via* nucleotide exchange and substrate release so that the Hsp70 chaperone can cycle on and off the aggregate surface (Kaimal et al., 2017). Intriguingly, yeast JDP Apj1 together with Hsp70 was shown to support disaggregation of intra-nuclear aggregates independent of Hsp104 (den Brave et al., 2020). In that line, bacterial Hsp70 was also shown to possess limited disaggregation activity, especially on large aggregates, (Ben-Zvi et al., 2004), thus it will be an exciting task to determine the mechanistic fundamentals how Hsp70s differ in their disaggregation capacities in different species.

Metazoans lack cytosolic/nuclear Hsp100 orthologs and thus rely on the disaggregation activity provided by Hsp70 itself in cooperation with a specific subset of JDP co-chaperones and NEFs from the Hsp110 family (simplified overview found in Figure 3). Together, these factors are capable of dissolving a wide range of aggregates *in vitro* and *in vivo* (Shorter, 2011; Rampelt et al., 2012; Mattoo et al., 2013; Nillegoda et al., 2017; Nillegoda et al., 2015; Gao et al., 2015; Nillegoda and Bukau, 2015; Kirstein et al., 2017). The promiscuity towards aggregated substrates is configured by different JDPs and the formation of mixed-class JDP complexes guarantees fine-tuning of the selection of aggregated proteins (Duennwald et al., 2012; Mattoo et al., 2013; Nillegoda et al., 2017; Nillegoda et al., 2015; Gao et al., 2015; Kirstein et al., 2017). The Hsp110 NEF enhances protein disaggregation activity and appears to function primarily by resetting the disaggregase machinery for new rounds of polypeptide extractions *via* substrate release. Hsp110 has also been proposed to take part in the disaggregation process by providing a holdase activity and hence to directly interact with substrate polypeptides (Mattoo et al., 2013; Gao et al., 2015; Nillegoda and Bukau, 2015; Nillegoda et al., 2015).

The Hsp70 machinery is central to all fates of resolubilized polypeptides. NEFs, driving the release of substrates, presumably play decisive roles in discrimination between refolding and degradation (Brehmer et al., 2001; Bracher and Verghese, 2015; Rosenzweig et al., 2019). As the Hsp70 disaggregase has inherent refolding activity, the machine is primed for efficient refolding after aggregate extraction and a handover mechanism to Hsp90 chaperones ensures a strong bias towards refolding over degradation (Nillegoda et al., 2018; Kohler and Andréasson, 2020). Yet, the integrated nature of the system with Hsp70 involved both in the disaggregation step as well as in downstream folding pathways has made it difficult study these separate functions. Likely, the nature of the substrate determines just how dependent its refolding is on Hsp70 following disaggregation. Based on the insight from studying *de novo* folding of proteins at ribosomes, small fast-folding substrates may fold completely without the aid of Hsp70 while more complex multidomain proteins are likely to depend of the chaperone (Komar, 2009; Kramer et al., 2009; Fedyukina and Cavagnero, 2011; Oh et al., 2011; Han et al., 2012).

Hsp70 was shown to protect stress granules from accumulation of misfolding-prone proteins like SOD1 and is required for dissolution of SOD1-containing stress granules that transitioned from liquid-like to solid-like state (Mateju et al., 2017). Further, the Hsp70 disaggregation machinery governs dissolution of stress granules containing defective ribosome products and associates with ribonuclear granules to maintain them in liquid-like states (Ganassi et al., 2016; Mateju et al., 2017). Similarly, yeast Hsp104 is essential for disaggregation of solid-like stress granules, originally assumed to be aggregates (Cherkasov et al., 2013; Kroschwald et al., 2015; Wallace et al., 2015; Riback et al., 2017; Franzmann et al., 2018). For example, this chaperone is required for the release of phase-separated Ded1 and other condensate-forming proteins like Pab1 from aged stress granules to facilitate re-entry into the cell cycle by starting translation of housekeeping changes during recovery after heat stress (Kroschwald et al., 2018; 2015; Riback et al., 2017; Yoo et al., 2022). Hsp104 and Hsp70 co-localize with stress granules, which is essential for post-stress recovery (Cherkasov et al., 2013).

Alternative disaggregation machineries like RuvBL1/2, Cyp40, HTRA1 or VCP/p97 have been discussed in recent years, some of them favoring degradation after extraction of misfolded proteins from aggregates, (Ratajczak et al., 1993; Hirabayashi et al., 2001; Tennstaedt et al., 2012; Poepsel et al., 2015; Zaarur et al., 2015; Baker et al., 2017; Darwich et al., 2020). RuvBL1/2 is described to exhibit a general, yet limited disaggregase activity and is proposed to function as chaperone in complex with Hsp90 to regulate assembly of nucleolar ribonucleoprotein complexes. RuvBL1/2 was shown to suppress seeding of amyloids and co-sediments with amyloid assemblies in human cells (Olzscha et al., 2011; Zaarur et al., 2015).

Perhaps of physiological relevance, disaggregation in metazoans appears to be slower than in yeast, as 5 h after heat shock most of the aggregates were still on their way to be fully disaggregated in human cells (Rampelt et al., 2012; Wallace et al., 2015; Määttä et al., 2020). Similarly, a *C. elegans* study showed that while there is a minute-scale disaggregation rate for misfolded luciferase *in vitro*, traces of luciferase aggregates were found days after heat shock *in vivo* (Kirstein et al., 2017). It should be noted in this regard that *in vitro* disaggregation models using firefly luciferase are very sensitive to the aggregation conditions employed in the experiments and not every type of aggregate can be disaggregated *in vitro*. Thus, it is likely that the cell offers an environment and factors that greatly impact on the physiological states of the aggregates and hence how efficiently they can be disaggregated. For example, orchestrated aggregation likely depends on LLPS as well as specific aggregation factors, for example, sHSPs, that ensure that the aggregates/condensates downstream are compatible with efficient and quantitative disaggregation (Mogk et al., 2019).

### 3.2 The sequential actions of disaggregation and degradation

**FIGURE 4:**
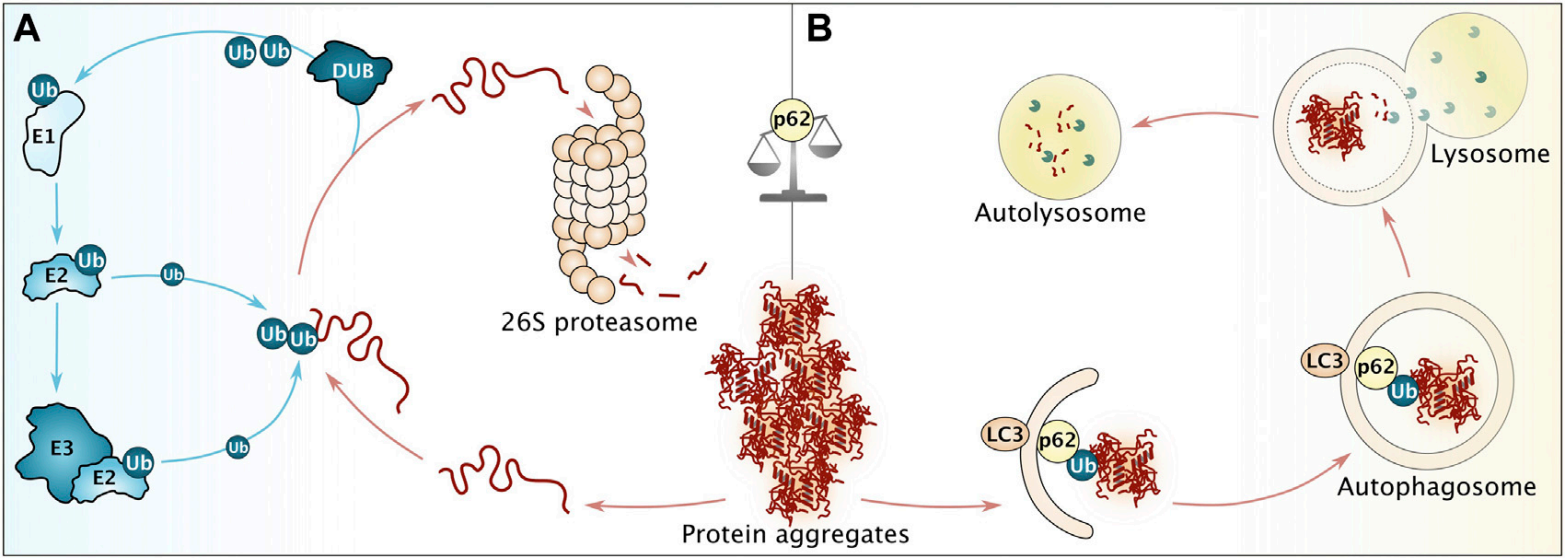
Overview of major cellular degradation pathways. **(A)** Before degradation *via* the ubiquitin-proteasome system (UPS), aggregated proteins require disaggregation, followed by decoration with poly-ubiquitin (Ub) moieties. This reaction is facilitated by ubiquitin-activating enzymes (E1), ubiquitin-conjugating enzymes (E2) and ubiquitin ligases (E3). Prior to degradation, ubiquitin moieties are removed by deubiquitinating enzymes (DUB). **(B)** Persistent aggregates are subjected towards autophagosomal degradation. To that end, p62 recognizing ubiquitin moieties facilitates the interaction of aggregates with LC3 on the growing autophagosome membrane. Please note that more proteins are involved to ensure efficient autophagy. After membrane closure, the autophagosome fuses with lysosomes in metazoan, leading to degradation of proteinaceous content (in autolysosomes). p62 (among other factors) serves to balance degradation *via* UPS and autophagy.

Terminally damaged or irretrievably misfolded proteins are extracted from aggregates and targeted for degradation *via* one of two dominant degradative pathways in cells, the ubiquitin-proteasome system (UPS) and autophagy (Figure 4) (Wang and Le, 2019). Similar to the scenario of refolding, UPS degradation of aggregates typically requires disentanglement of the substrate by disaggregation. The scenario of autophagy may or may not involve such previous disaggregation depending on if the pathway involved belongs to macro- or microautophagy (Saha et al., 2018; Wang et al., 2022). Chaperone-mediated autophagy, a selective form of microautophagy relying on the Hsp70 system for its specificity, takes care of soluble substrates and has been described only in metazoan cells so far (reviewed in (Kaushik and Cuervo, 2018)).

Metazoan VCP/p97 (homologous to yeast and plant Cdc48) and ATP-independent HTRA1 can execute disaggregation (Nillegoda et al., 2018). Human HTRA1 is a serine protease and is implicated in diseases involving proteostasis imbalances and was shown to efficiently degrade neurodegenerative proteins Aβ and Tau, *in vitro* and *in vivo*. Furthermore, protease-inactivated HTRA1 converts fibrillar tau to soluble species, substantiating its disaggregation activity (Wilken et al., 2004; Yang et al., 2006; Tennstaedt et al., 2012; Poepsel et al., 2015). Interestingly, the combination of disaggregation and immediate proteolytic degradation of substrates in HTRA1 functionality, guarantees uni-directionality of this process, thus elimintes the requirement of ATP-dependent substrate release. The generation of short peptides with proposedly lower affinity to respective binding sites, ensures the energy-independent dissociation of degraded substrates from HTRA1 (Poepsel et al., 2015).

Another disaggregase system tightly associated with subsequent degradation is VCP/p97 belonging to the AAA+ superfamily. It consists of a hexameric ring, coupling ATP hydrolysis with mechanical work. Its structural similarity to yeast Hsp104 and bacterial ClpB made it an early focus of interest when hunting for metazoan disaggregase machineries (Parsell et al., 1994; Hirabayashi et al., 2001; Kobayashi et al., 2007). In contrast to HTRA1, VCP/p97 collaborates with the UPS for proteolysis of its substrates. VCP/p97 is involved in PQC, ribosome-associated quality control as well as ER-associated degradation (ERAD) and has been shown to reduce cytotoxicity associated with polyQ aggregates in *Drosophila melanogaster* and *C. elegans* (Ballar et al., 2011; Bodnar and Rapoport, 2017; Defenouillère et al., 2013; Nishikori et al., 2008; Verma et al., 2013). It further plays a role in aggregate clearance from the nucleus after heat shock and engages with tau and huntingtin in *post-mortem* brain tissue (Hirabayashi et al., 2001; Gallagher et al., 2014; Darwich et al., 2020). Thus, cells harbor machineries that directly link disaggregation to degradation using specialized proteases or the UPS system.

UPS targets the majority of cellular proteins and ubiquitin conjugation is facilitated by an enzymatic cascade, modulating protein half-lives from seconds to days (Figure 4A) (Galves et al., 2019). Ubiquitin is activated by ubiquitin-activating enzymes (E1), handed over to ubiquitin-conjugating enzymes (E2) and transferred either directly onto the substrate or *via* ubiquitin ligases (E3). Prior to degradation, ubiquitin is removed by deubiquitinating enzymes (DUB) (Hoeller et al., 2007; Pohl and Dikic, 2019). Ubiquitinated proteins are recognized and degraded by the 26S proteasome holoenzyme, consisting of a barrel-shaped 20S core particle with proteolytic activity, capped on both ends by 19S regulatory particles, responsible for recognition and regulatory processes (Lu et al., 2017; Coleman et al., 2021).

The function of the proteasome has been suggested to be regulated at the subcellular level. Its localization is highly dynamic, most recent studies point towards predominant nuclear localization in metazoan, similar as observed in yeast (Lehmann et al., 2002; Marshall et al., 2016; Nemec et al., 2017; Tomita et al., 2019). Interestingly, starvation and nutrient depletion in plants lead to proteasome disassembly and thus inactivation into storage granules (Gu et al., 2017). VCP/p97, proteasome particles, E1-, E2-and E3-enzymes as well as DUBs are found in biomolecular condensates in the nucleus and p62, an adaptor protein linking UPS and autophagy, was shown to be an essential component of these droplets (Fu et al., 2021). LLPS properties is brought on by p62 self-interaction as well as interaction with poly-ubiquitin chains (Sun et al., 2018; Zaffagnini et al., 2018). These foci have been proposed to function as catalytic centers for degradation of misfolded proteins that are assembled by proteotoxic cues, for example, heat and oxidative stress. The droplets are likely only acceptable under transient stress conditions, since prolonged engagement of disaggregases and proteasomes would hinder them from performing their canonical roles in proteostasis. Empirically, inhibition of VCP/p97 has been shown to increase foci size, while suppression of ubiquitination activity prevents foci formation (Yasuda et al., 2020). Also stress granules show a related behavior. Ubiquitinated proteins, DUBs and several ubiquitin-binding proteins have been shown to accumulate in these condensates, with DUBs adopting a regulatory role in stress granule dynamics. The VCP/p97 disaggregation machinery contributes to clearing stress granules and mutations of VCP/p97 delay disappearance of stress granules and cellular recovery (Kwon et al., 2007; Buchan et al., 2013; Ganassi et al., 2016; Kedersha et al., 2016; Mateju et al., 2017; Dao et al., 2018; Turakhiya et al., 2018; Xie et al., 2018; Youn et al., 2018; Wang et al., 2019). Interestingly, cytosolic stress granules were shown to relieve the nuclear UPS by trapping misfolding-prone defective ribosomal products *via* LLPS and thus hindering their accumulation in the nucleus (Xu et al., 2022).

Proteins that fail to undergo efficient disaggregation remain as large and persistent protein aggregates and are ultimately directed towards autophagy for bulk destruction (Figure 4B). This self-eating mechanism involves *de novo* membrane synthesis around the aggregate which results in engulfment of the cargo into autophagosomes, followed by their subsequent delivery to and fusion with proteolytic lysosomes (autolysosomes) and involves a plethora of receptors and other regulatory proteins (Melentijevic et al., 2017; Runwal et al., 2019). Targeting works *via* cargo recognition by adaptor proteins with p62 (Cue5 in yeast) playing a key role by interacting with ubiquitin moieties on the protein aggregates, and linking them to LC3 (or Atg8 in yeast) on the autophagosome membranes. Selective degradation of aggregates *via* autophagy (aggrephagy) plays a critical role in limiting their accumulation in cells (Kraft et al., 2009; Sun et al., 2020). Stress granules associated with ubiquitinated proteins recruit p62, thus triggering degradation of ubiquitin-positive stress granules by autophagy (Ganassi et al., 2016; Mateju et al., 2017). Thus, cells are equipped with regulatory mechanisms, ensuring the beneficial role of stress granules on the UPS system as dumping ground for aggregation-prone proteins but at the same time prompting their removal, if these condensates become oversaturated with ubiquitinated proteins.

Intriguingly, there is a substantial crosstalk between the UPS and autophagy. In this regard, p62 orchestrates the UPS and autophagy with a mechanistic base in its innate ability to physically associate with both systems. It either escorts ubiquitinated proteins to the proteasome as shuttling factor or to autophagosomes as autophagy receptor (Seibenhener et al., 2004; Pankiv et al., 2007; Wurzer et al., 2015). Autophagy is upregulated in conditions of deficient degradation of ubiquitinated proteins by UPS, either *via* genetic or pharmacological proteasome inhibition (Pandey et al., 2007; Wu et al., 2008; Zhu et al., 2010; Laussmann et al., 2011; Selimovic et al., 2013; Li et al., 2019). Furthermore, loss of Hsp70 or its NEF Hsp110 also reduces proteasome activity and simultaneously induces autophagy (Feleciano et al., 2019). Proteasome inhibition leads to its ubiquitination in yeast, initiating the first steps towards selective degradation of inactivated proteasomes *via* autophagy (Marshall et al., 2016; Waite et al., 2016; Nemec et al., 2017). Crosstalk also goes the other way if UPS gets overwhelmed. In that scenario, misfolded or damaged proteins form large insoluble aggregates that cannot rapidly be removed by the proteasome, thus requiring degradation *via* autophagy (Hyun et al., 2003). Moreover, protein aggregates were shown to inhibit the proteasome, also requiring the activation of autophagy (Bence et al., 2001; Lindersson et al., 2004; Díaz-Hernández et al., 2006; Myeku et al., 2016; Sun-Wang et al., 2020). In summary, the UPS and autophagy complement each other and share both substrates and common factors.

### 3.3 Interplay between refolding and degradation

Proteins that adopt an aberrant fold are rapidly recognized by chaperones that prevent aggregation, facilitate folding, and coordinate the interaction with E3 ubiquitin ligases, thus coupling protein refolding with degradation. The chaperone machinery interacts only transiently with the aberrantly folded substrate, yet it will rebind in repeated cycles if the protein does not reach its native state and remains misfolded (Kohler and Andréasson, 2020). The key chaperone Hsp70 employs NEFs that ensure efficient release and thus cycling in the chaperone network (Rosenzweig et al., 2019; Kohler and Andréasson, 2020). For persistently misfolded proteins, release from Hsp70 is mediated by dedicated armadillo and BAG-type NEFs that carry specialized substrate release domains that compete the substrate off the chaperone substrate binding site (Rauch et al., 2016; Gowda et al., 2018; Rosam et al., 2018). This orchestrated release enables other factors such as ubiquitin E3 ligases to get access to the hydrophobic peptides that constitute the Hsp70 binding site and also function as degrons (Rauch et al., 2016; Gowda et al., 2018; Rosam et al., 2018; Abildgaard et al., 2023). For example, the metazoan E3 ligase CHIP associates with Hsp70 and will eventually ubiquitinate persistently misfolded proteins that undergo repeated Hsp70 interaction cycles (Murata et al., 2001). CHIP mediates substrate ubiquitination through its C-terminal catalytic domain and its N-terminus interacts with Hsp70. The interaction with the disaggregation machinery is essential for CHIP activity, as aggregate-bound chaperones are required for efficient ubiquitination (Qian et al., 2006; Tetzlaff et al., 2008; Kalia et al., 2011). Whether misfolded proteins are subjected towards refolding or degradation has been suggested to depend on the ratio between disaggregation machinery and CHIP. Elevated CHIP expression and prolonged Hsp70-substrate interaction lead to a larger fraction of substrates becoming ubiquitinated by CHIP and thus targeted for degradation (Qian et al., 2006). Hsp70 and other chaperones are expressed at significantly higher levels than CHIP, suggesting a potential cellular preference for refolding over degradation (Meacham et al., 2001). Regulation of proteasomal targeting of Hsp70-disentangled substrates is also assisted by the NEF Bag1, that carries a ubiquitin-binding domain for specific recognition of modified substrates as well as Hsp70 substrate release domains (Lüders et al., 2000; Tsukahara and Maru, 2010; Rauch et al., 2016; Hantouche et al., 2017). In line, BAG3:BAG1 ratio determines degradation *via* UPS or autophagy. Proteotoxic stress and cellular ageing lead to BAG3 upregulation and outcompete BAG1, redirecting Hsp70-CHIP-associated substrates towards autophagy-mediated degradation pathways (e.g., chaperone-assisted selective autophagy) (Carra et al., 2008; Gamerdinger et al., 2011; Gamerdinger et al., 2009; Minoia et al., 2014; Rapino et al., 2014). A related role is played by Hsp70 NEFs of the Hsp110 class that interact with the proteasome as well as with Hsp70 as a substrate releasing NEF. Thus, Hsp110 functions as an Hsp70-misfolded protein receptor at the proteasome that fast-tracks misfolded protein associated with Hsp70 for degradation and ensures the timely release from the chaperone close to the proteolytic chamber (Kandasamy and Andréasson, 2018; Gersing et al., 2021). These functions appear to be conserved between yeast and man (Rauch et al., 2016). The intricate interdependency between Hsp70-dependent refolding, aggregate sequestration by sHsps and the UPS has been recently tested by genetic analysis in yeast. Imbalances between the activity systems rather than the processes themselves were found to severely affect protein homeostasis and cellular fitness (Jawed et al., 2022).

When assessing the ratio between refolding and degradation rates, PQC has a preference towards rescuing misfolded proteins with only little degradation of reporter and bulk proteins, as observed both in yeast and in mammalian cell culture during recovery from mild cellular stresses (Hageman et al., 2007; Medicherla and Goldberg, 2008; Wallace et al., 2015; Määttä et al., 2020). Refolding of aggregated proteins spares the cell the burden of novel biosynthesis of metabolic and regulatory proteins, which is vital to rapid recovery of cellular functions after stress (Medicherla and Goldberg, 2008). However, the interrelation of refolding and degradation networks ensures that the occurrence and persistence of proteotoxic species is prevented by all means, even if one subsystem malfunctions.

### 3.4 PQC failure due to disease

Increasing age decreases the functionality of PQC, resulting, e.g., from lower expression and/or activity of proteasome subunits and ubiquitin-related enzymes, and thus leads to occurrence and accumulation of toxic protein aggregates (Lee et al., 2000; Lee et al., 1999; Ly et al., 2000; Petropoulos et al., 2000; Ferrington et al., 2005; Tonoki et al., 2009). Mutations in genes encoding for chaperones and PQC-related factors further accelerate the decline leading to age-associated disorders (H et al., 2022; Macario et al., 2005; Tittelmeier et al., 2020), as has been seen for mutations in VCP/p97 that are linked to neurodegenerative disorders (Watts et al., 2004; Johnson et al., 2010; Meyer and Weihl, 2014). Similarly, alterations of degradation pathways are linked to the onset of human age-related diseases including cancer and neurodegeneration. Additionally, autophagy is shown to be dysfunctional in neurodegenerative diseases and loss-of-function mutations in the autophagy machinery leads to an early onset of neurodegenerative phenotypes in mice (Hara et al., 2006; Komatsu et al., 2006; Corti et al., 2020; Rana et al., 2021). Neurodegenerative disorders such as Alzheimer’s disease, Parkinson’s disease and Huntington’s disease involve loss of proteasomal degradation activity (Keller et al., 2000; Furukawa et al., 2002; McNaught et al., 2002; Seo et al., 2004; Díaz-Hernández et al., 2006; Thibaudeau et al., 2018), while UPS-associated enzymes, ubiquitin moieties and chaperones are abundantly detected in insoluble aggregate structures such as Lewy bodies and neurofibrillary tangles, which further underpins the abortive attempt of PQC to counteract pathological aggregation processes (García-Sierra et al., 2012; Zhang et al., 2019; Arakhamia et al., 2020; Schweighauser et al., 2020).

## 4 Outlook and open questions

The strict distinction between biomolecular condensates as easily reversible and beneficial vs. aggregates as irreversibly, toxic compartment is not reflecting the fact that aging condensates can adopt solid-like, irreversible properties, while especially protein-disorder diseases show that aggregation processes producing almost inert compartments can indeed be the less toxic species compared to soluble oligomers. Thus, a more nuanced way of interpretation is required. Especially substantiated by the fact that distinction between phase-separated condensates and aggregates formed by misfolded protein species is not always the easiest task, as seen in yeast, where stress granules were originally identified as aggregated compartments. However, as discussed above, biomolecular condensates can incorporate misfolded proteins and it is hypothesized that phase separation might be a transition state preceding aggregate formation. Traditionally, LLPS has been widely studied *in vitro* under controlled and predictable conditions, a valuable tool gathering basic knowledge on these processes, nevertheless, *in vivo* data reflecting the dynamic nature of this process is inevitable for a complete picture. The facts that biomolecular condensates can adopt different states ranging from liquid-like to solid-like, harbor different scaffold and client proteins depending on cellular conditions and govern essential cellular processes make their full potential hard to understand. Research has just begun to explore aging as additional complexity factor, as traditionally PQC studies, especially those in model systems like yeast and bacteria, were conducted in young, immaculately working systems that instantly react to proteotoxic cues to safeguard cellular proteostasis. In light of our perpetually aging society and the fact that PQC capacity decreases over time (Hipp et al., 2019), it is vital to understand the parallels and differences between young and aged proteostasis systems. We already know that several PQC factors, including the proteasome, loose their competence over time. Interestingly, while some organisms develop age-associated diseases like cancer and neurodegeneration, others remain healthy. Not taking heritability and external triggers into account, it might additionally be feasible that other PQC branches take over to guarantee a balanced and healthy proteome. While rapidly-growing cells are especially challenged by the flood of ribosomal products prone to misfolding and subsequent aggregation, non-dividing cells like neurons or modelled by stationary post-diauxic yeast supposedly face different problems. As nuclear-localized proteins involved in DNA binding, chromatin organization and transcriptional activity were recently shown to be especially vulnerable to heat shock, substantiated by the fact that topoisomerases and proteins involved in DNA replication had the lowest melting points in bacteria (Mateus et al., 2018; Määttä et al., 2020), it seems intuitive that PQC forces need to be consolidated to counteract sudden proteotoxic impact protecting the weakest members of the cellular proteome. Thus, it is feasible that specific PQC components alter their localization or expression patterns with increasing age. While several studies focused on gene expression upon proteotoxic cues and age, especially potential distribution patterns of major chaperones and folding factors represent exciting research questions for future studies.
